# Smart Layer-by-Layer Polymeric Microreactors: pH-Triggered Drug Release and Attenuation of Cellular Oxidative Stress as Prospective Combination Therapy

**DOI:** 10.1021/acsami.1c01450

**Published:** 2021-04-16

**Authors:** Edurne Marin, Neha Tiwari, Marcelo Calderón, Jose-Ramon Sarasua, Aitor Larrañaga

**Affiliations:** † Department of Mining-Metallurgy Engineering and Materials Science, POLYMAT, Faculty of Engineering in Bilbao, University of the Basque Country (UPV/EHU), Plaza Torres Quevedo 1, 48013 Bilbao, Spain; ‡ POLYMAT, Applied Chemistry Department, Faculty of Chemistry, University of the Basque Country UPV/EHU, Paseo Manuel de Lardizabal 3, 20018 Donostia-San Sebastian, Spain; § IKERBASQUE, Basque Foundation for Science, 48009 Bilbao, Spain

**Keywords:** oxidative stress, polymer capsules, layer-by-layer, multifunctional vehicles, drug release

## Abstract

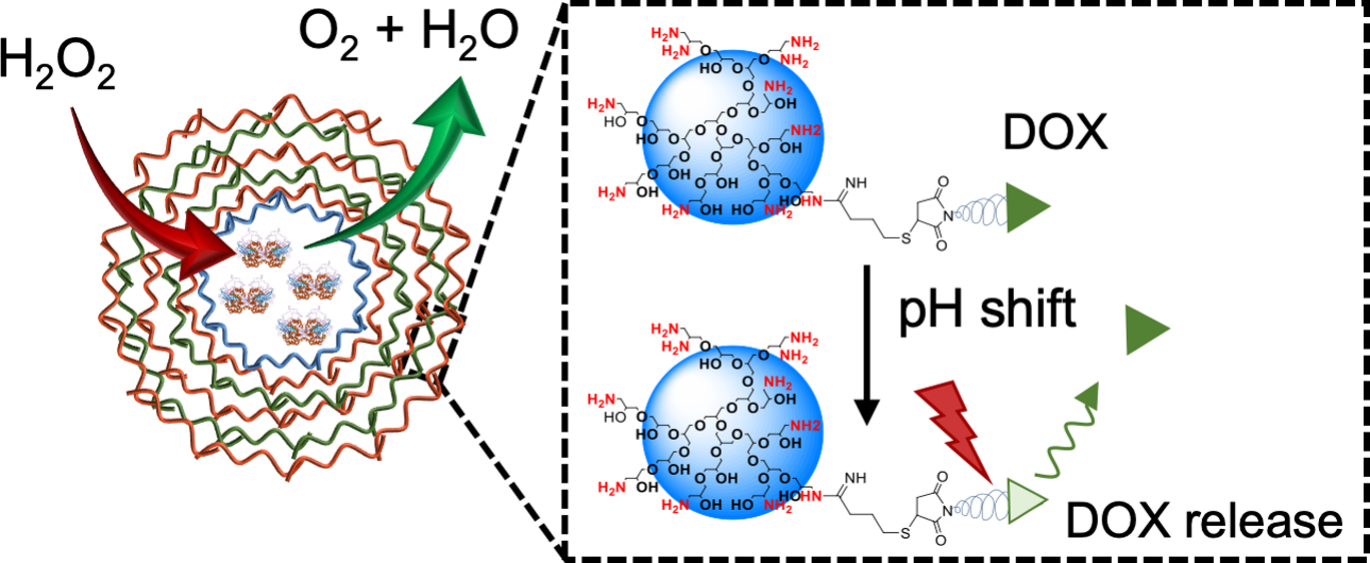

Polymer capsules fabricated *via* the layer-by-layer (LbL) approach have emerged as promising biomedical systems for the release of a wide variety of therapeutic agents, owing to their tunable and controllable structure and the possibility to include several functionalities in the polymeric membrane during the fabrication process. However, the limitation of the capsules with a single functionality to overcome the challenges involved in the treatment of complex pathologies denotes the need to develop multifunctional capsules capable of targeting several mediators and/or mechanisms. Oxidative stress is caused by the accumulation of reactive oxygen species [*e.g.*, hydrogen peroxide (H_2_O_2_), hydroxyl radicals (^•^OH), and superoxide anion radicals (^•^O_2_^–^)] in the cellular microenvironment and is a key modulator in the pathology of a broad range of inflammatory diseases. The disease microenvironment is also characterized by the presence of proinflammatory cytokines, increased levels of matrix metalloproteinases, and acidic pH, all of which could be exploited to trigger the release of therapeutic agents. In the present work, multifunctional capsules were fabricated *via* the LbL approach. Capsules were loaded with an antioxidant enzyme (catalase) and functionalized with a model drug (doxorubicin), which was conjugated to an amine-containing dendritic polyglycerol through a pH-responsive linker. These capsules efficiently scavenge H_2_O_2_ from solution, protecting cells from oxidative stress, and release the model drug in acidic microenvironments. Accordingly, in this work, a polymeric microplatform is presented as an unexplored combinatorial approach applicable for multiple targets of inflammatory diseases, in order to perform controlled spatiotemporal enzymatic reactions and drug release in response to biologically relevant stimuli.

## 1 Introduction

The layer-by-layer (LbL) technique is a simple and versatile method that allows the modification of a wide variety of substrates (*e.g.*, planar structures, fibers, and colloidal particles) through the alternate deposition of oppositely charged polyelectrolytes.^1−4^ This technique has evolved from the application on planar substrates to colloidal micro- and nanoparticles in the late 1990s thanks to the intensive investigations by Möhwald and collaborators.^5−8^ In these pioneering studies, highly charged polyelectrolytes were deposited onto colloidal particles by taking advantage of their stability, selectivity, and permeability.^7,9^ The colloidal core was subsequently removed, giving rise to hollow polymer capsules.

Recent progresses in the field of bioscience and polymer synthesis allow the fabrication of polymer capsules using alternative biodegradable synthetic and natural polymers, proteins, or inorganic nanoparticles, among others, their properties being tunable for specific biomedical requirements.^2,8,10−12^ Thus, taking advantage of this versatility and capability to fabricate polymer capsules with tailor-made properties, capsules have been fabricated for a wide variety of applications, including drug/protein/gene delivery vehicles,^13−16^ polymer capsules for imaging applications,^17−19^ or micro- and nanoreactors.^12,20−22^ The latest contain active entities in their core and allow the diffusion of reagents and byproducts through the polymer shell. The active compounds [*e.g.*, enzymes or nanozymes^23^ (*i.e.*, synthetic nanomaterials with enzyme-like characteristics)] are protected from the outer microenvironment and act *in situ*.^1,20^

However, in most of these applications, capsules are endowed with a single functionality, which may limit their potential to overcome the challenges involved in the treatment of complex pathologies and to adapt to patient-specific characteristics.^24^ This denotes the need to develop multifunctional capsules, which respond to different physiological stimuli and adjust to the individual particularities of the patient.^11,24,25^ Excellent examples of such multifunctional capsules are theranostic micro- and nanocapsules, which are capable of simultaneously diagnosing and treating the damaged site, while acting also as imaging agents.^26−30^ To impart these advanced functionalities, the polyelectrolytes employed for the fabrication of polymeric membranes can be modified, incorporating several functionalities and (bio)molecules (*e.g.*, drugs, antibodies, or proteins) which will respond to specific external or local stimuli.^1,29^

The deconstruction of the capsule is usually required for the efficient triggered delivery of the encapsulated therapeutic agent. Either internal (*i.e.*, local) or external stimuli can facilitate the disruption of the capsule by different mechanisms. For example, a decrease in pH (*e.g.*, mimicking endosomal pH conditions) causes charge repulsion between the polyelectrolytes, leading to the rapid release of the encapsulated cargo.^31^ Decorating the polymeric membrane with magnetic- (*e.g.*, iron oxide nanoparticles^32^), ultrasound- (*e.g.*, gold nanoparticles^14^), and near-infrared-responsive (*e.g.*, graphene oxide^33^) nanoparticles allows the disassembly of the polymeric shell and the subsequent release of the encapsulated therapeutic agent. All these strategies are clearly inappropriate when microcapsules are intended to be used as microreactors. Ideally, for the application we intend to pursue, the polymeric capsule should maintain its structural integrity when the complementary drug is released to ensure the protection of the encapsulated enzyme.

In our approach, the use of dendritic polyglycerols (dPGs) is envisioned as an unexplored strategy to impart additional functionalities to the capsules while preserving their structural integrity. Dendritic polymers present a high solubility, biocompatibility, and a high functionality.^34,35^ Hence, a wide variety of active compounds, such as bioactive molecules or targeting moieties, can be conjugated to the dPG branches using cleavable bonds which will respond to the stimuli and specific conditions of the damaged area (*e.g.*, acidic pH, overexpressed enzymes, or reducing media).^34−37^

When the native cellular regulation of reactive oxygen species (ROS) production [*e.g.*, hydrogen peroxide (H_2_O_2_), hydroxyl radicals (^•^OH), superoxide anion radicals (^•^O_2_^–^)] is overwhelmed, oxidative stress, which is implicated in numerous pathologies such as neurodegeneration, cancer, osteoarthritis, or cardiovascular diseases, occurs.^38−40^ Furthermore, oxidative stress is usually accompanied by dysregulated inflammatory responses and a reduction in the environmental pH.^40−42^ Thus, to overcome the complexity of an oxidative stress microenvironment, multifunctional biomedical systems mentioned above will be of great interest.

In this study, it was hypothesized that the LbL approach, in combination with dPG–drug conjugates, could be exploited to create multifunctional polymer capsules capable of simultaneously reducing the levels of ROS while releasing a model drug in response to a biologically relevant stimulus (*i.e.*, pH). We fabricated multifunctional polymer capsules by depositing alternate layers of poly(sodium 4-styrenesulfonate) (PSS), poly(allylamine hydrochloride) (PAH), and an amine-containing dPG conjugated to doxorubicin (dPG–DOX), which was employed as a model drug to test the potential of the system, on a CAT-loaded CaCO_3_ sacrificial template. DOX was conjugated to dPG through a pH-responsive linker. After the removal of the CaCO_3_ template, multifunctional capsules were obtained. The physicochemical, morphological, and functional properties of the capsules were thoroughly determined. A preliminary *in vitro* model of oxidative stress with HeLa cells was used to assess the therapeutic potential of the capsules.

## 2 Results and Discussion

### 2.1 Synthesis of dPG–DOX Conjugate

**Figure 1:**
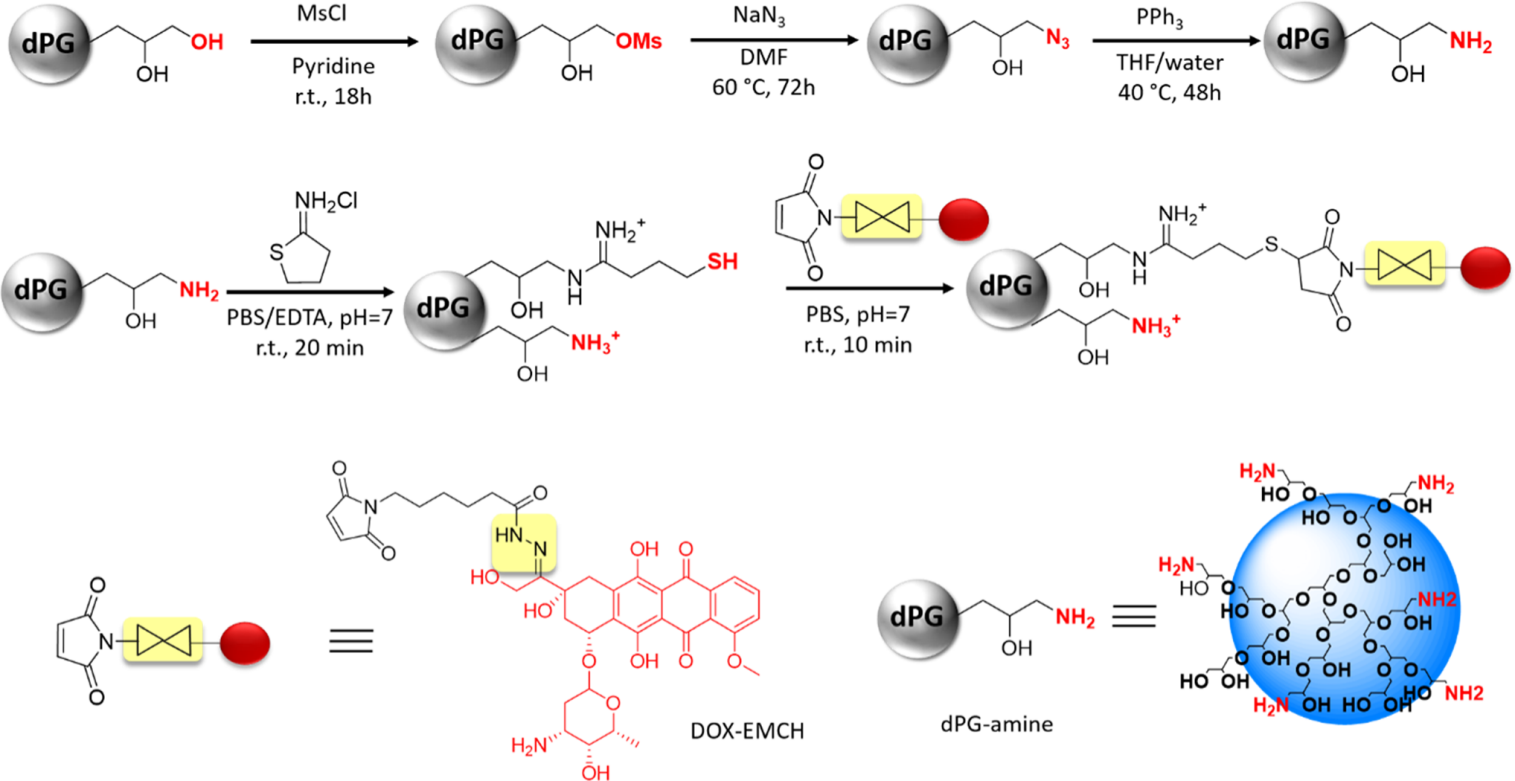
Reaction pathways for the synthesis of dPG-amine and dPG–DOX conjugate. The depicted structure of dPG-amine represents only a fraction of the total polymer.

Polyglycerol amine (dPG-amine) was synthesized, following a three-step protocol. The −OH groups of dPG were first converted to mesyl (Ms) groups, followed by the conversion of Ms groups to azide functionalities. The azide groups were subsequently transformed into amine groups using triphenylphosphine as the reducing agent. Figure 1 shows the schematics and the reaction conditions for the synthesis of dPG-amine from dPG as the starting material. All samples were well characterized using nuclear magnetic resonance (NMR) spectroscopy (Figures S1–S3, Supporting Information), and a total of 15 mol % amine grafting was obtained (*i.e.*, 18 NH_2_ and 103 OH groups, on average). The conversion of azide groups to amine moieties was further confirmed by the disappearance of the characteristic peak of azide groups at 2100 cm^–1^ after reduction reaction (Figure S4, Supporting Information). The zeta potential of dPG-amine was 12 ± 2 mV, confirming the presence of amine functionalities on dPG moieties. The hydrodynamic sizes of dPG-amine were found to be around 20–25 nm with a high polydispersity index (PDI = 0.7), owing to the aggregation of the particles in solution.

The conjugate of dPG–DOX was synthesized through a one-pot synthesis, as stated in earlier publications.^36,43^ A schematic representation of the conjugation reaction between DOX–EMCH (*i.e.*, DOX bearing a pH-cleavable hydrazone bond, with a maleimide group for conjugation) and dPG-amine containing 15 mol % amino groups is shown in Figure 1. First, dPG-amine was thiolated to yield, on average, one thiol per dPG molecule. After thiolation, DOX–EMCH was added to the dPG–thiol solution to allow the reaction between the thiols and the maleimide groups through a selective Michael-type addition. After the reconstitution of the lyophilized samples in PBS buffer, the concentration of DOX was determined photometrically using the molar absorption coefficient of DOX at 495 nm. The first assessment of the pH-triggered cleavage and DOX release was performed by dispersing the dPG–DOX conjugate in sodium acetate buffer at pH 4.0 and applied on a G-25 Sephadex column. The amount of DOX conjugated to the dPG backbone was calculated to be ∼2 wt % or one DOX per three molecules of dPG using UV/vis spectroscopy at 495 nm (ε 495 = 10,645 M^–1^ cm^–1^). It should be noted that such rather low drug loading was aimed in order to enable the predominance of −NH_3_^+^ at the surface of the conjugates for their further incorporation into the microcapsules during the LbL deposition process. The hydrodynamic sizes of the conjugates remained in the same size range as dPG-amine. The zeta potential of dPG–DOX was 9 ± 0.5 mV, confirming the presence of amine functionalities on dPG moieties incorporating an overall positive charge on dPG even after DOX conjugation.

### 2.2 Fabrication of Polymer Capsules *via* the LbL Approach

**Figure 2:**
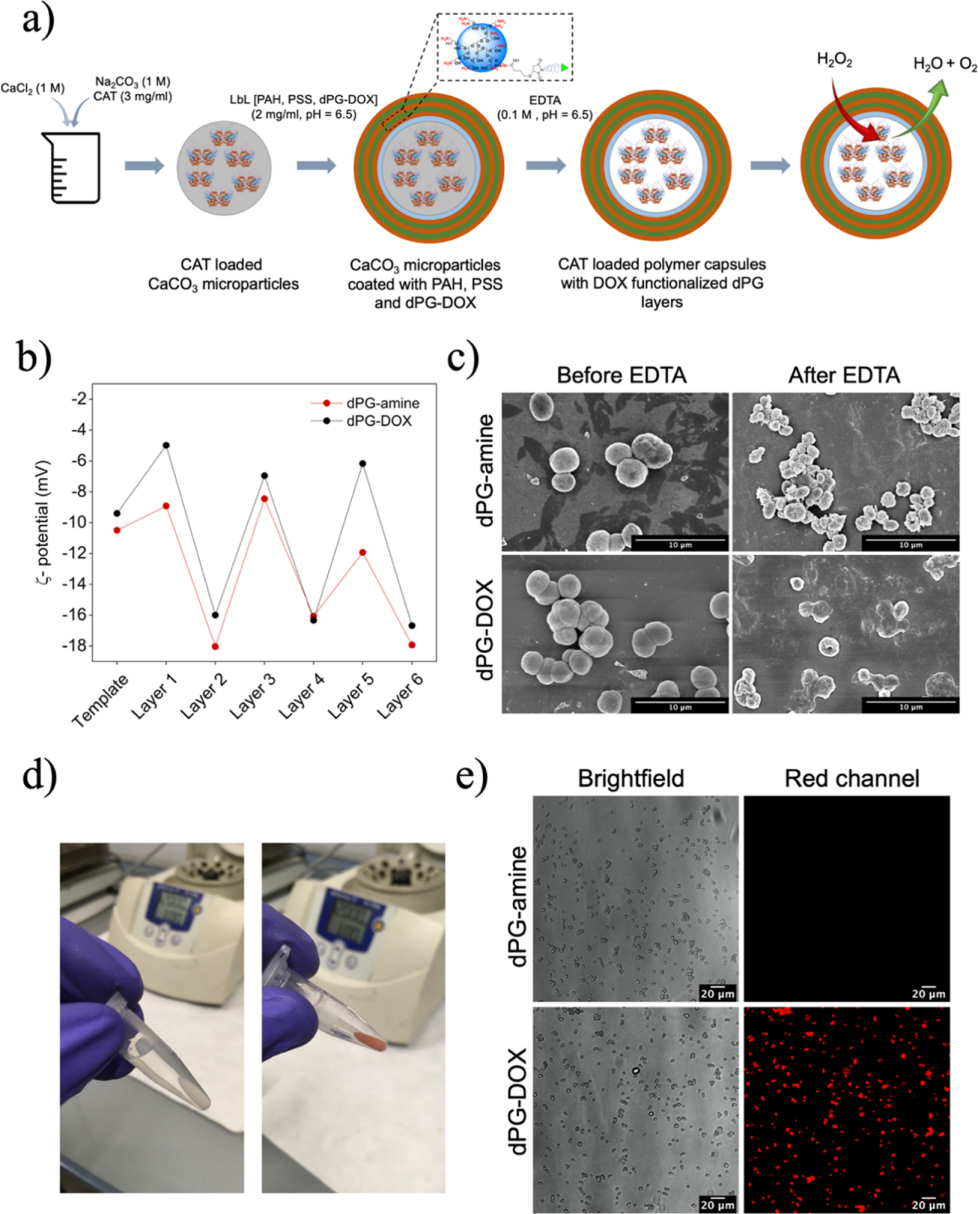
(a) Schematic representation of the fabrication of multifunctional polymer capsules, (b) ζ-potential of (PAH/PSS) (dPG–DOX/PSS)_2_ and (PAH/PSS) (dPG-amine/PSS)_2_ polymer capsules, (c) SEM micrographs of polymer capsules, (d) polymer capsules before (left) and after (right) incubation with dPG–DOX, and (e) fluorescence micrographs of capsules fabricated with dPG-amine or dPG–DOX.

For the fabrication of polymer capsules, CaCO_3_ sacrificial template was first synthesized through the co-precipitation of CAT, CaCl_2_, and Na_2_CO_3_ (Figure 2a) because of the reported higher encapsulation efficiency in comparison to alternative methods.^44,45^ The process resulted in CAT-loaded CaCO_3_ spherical microparticles with a mean diameter of 3.9 ± 1.6 μm, which slightly differed from the CaCO_3_ sacrificial template without the enzyme (3.1 ± 1.2 μm) (Figure S5, Supporting Information). CaCO_3_ microparticles were selected as the sacrificial template as they are easily dissolved using a calcium chelating agent [*i.e.*, ethylenediaminetetraacetic acid disodium salt dehydrate (EDTA)] and enable to avoid harsh conditions (*i.e.*, organic solvents and/or extremely high/low pH) in the subsequent template removal step, thus protecting the integrity of polyelectrolytes, the encapsulated enzyme, and the chemically conjugated drug.^46^ Contrary to other templates such as polystyrene beads^47^ and melamine formaldehyde cores,^48^ the protocol used herein allows the pre-encapsulation of the enzyme. Post-encapsulation of (bio)macromolecules in templated LbL capsules usually relies on an increased permeability of the polymeric membrane promoted by a change in solvent composition,^49^ pH,^50^ or temperature.^51^ These conditions could have a detrimental effect on the conformational integrity of the encapsulated enzyme, thus jeopardizing its catalytic activity.

Capsules containing six layers were fabricated using PAH as the first positive layer and dPG–DOX or dPG without the conjugated drug (dPG-amine) for the subsequent positive layers (Figure 2a), resulting in (PAH/PSS) (dPG–DOX/PSS)_2_ and (PAH/PSS) (dPG-amine/PSS)_2_ architectures, respectively. PAH and PSS polyelectrolytes were chosen due to their extensive use as model polyelectrolytes in the fabrication of LbL capsules and their robust structure enabling the transfer of substrates across the polymeric membrane, while protecting the active compound from the external environment.^46,52−54^ Although LbL capsules based on biodegradable polyelectrolytes are of high interest in several biomedical applications (*e.g.*, nanovehicles for the transfer of genetic material^55^ or drugs^56^), biodegradability may be an undesirable property when capsules are intended to be used as microreactors. If the degradation process is not carefully controlled, the encapsulated enzyme would be exposed to the external physiological conditions and suffer protease degradation and denaturation.

The sacrificial template was initially negatively charged (pI_CAT_ = 5.4). Therefore, CAT-loaded CaCO_3_ microparticles were first incubated with PAH, resulting in a shift in their surface charge from −9.4 ± 0.6 to −5.0 ± 0.3 mV in the case of capsules fabricated using dPG–DOX (Figure 2b) and from −10.5 ± 0.3 to −8.9 ± 1.2 mV in the case of capsules fabricated using dPG-amine (Figure 2b). Afterward, polyelectrolyte layers were assembled alternately, and a change in the ζ-potential value was observed after each deposition step, confirming the successful layer assembly (Figure 2b).

After the LbL process, the microparticles were immersed in 0.1 M EDTA solution to allow the removal of the CaCO_3_ sacrificial template (Figure 2a). As observed in scanning electron microscopy (SEM) micrographs, the fabricated capsules before EDTA addition had a spherical shape with a mean diameter size ∼3–4 μm (Figure 2c). After EDTA incubation, capsules fabricated with dPG–DOX and with dPG-amine were hollow, as suggested by their collapsed shape (Figure 2c). Fourier transform infrared spectra (FTIR) (Figure S6, Supporting Information), where the two main bands associated to CaCO_3_ at 1384 and 870 cm^–1^ disappeared after the incubation with EDTA, confirmed the successful elimination of the sacrificial template.

To test their stability, microcapsules were immersed in PBS at 37 °C, and micrographs were acquired at different time points (4, 24, and 72 h). As observed in SEM, capsules maintained their spherical and collapsed shape at the selected time points, confirming their stability over time (Figure S7, Supporting Information).

The successful adsorption of DOX was quantitatively and qualitatively assessed. After the incubation of the capsules with dPG–DOX, a change in the color of the microcapsule pellet was observed (Figure 2d). The successful incorporation of dPG–DOX was quantitatively confirmed by measuring the concentration of dPG–DOX in the supernatant before and after each layer deposition. The obtained results showed a decrease in the dPG–DOX concentration in the solution after each incubation, confirming the adsorption of the drug conjugate onto the capsules (Figure S8, Supporting Information). The adsorbed quantity of DOX was 1.9 μg per 10 mg of CaCO_3_ sacrificial template. Finally, the presence of DOX was qualitatively confirmed by means of fluorescence microscopy after the template removal. Red fluorescence was observed in the case of the capsules fabricated with dPG–DOX, whereas capsules fabricated with dPG-amine were not visible (Figure 2e).

### 2.3 Antioxidant Capacity and pH-Dependent Drug Release of the Polymer Capsules

**Figure 3:**
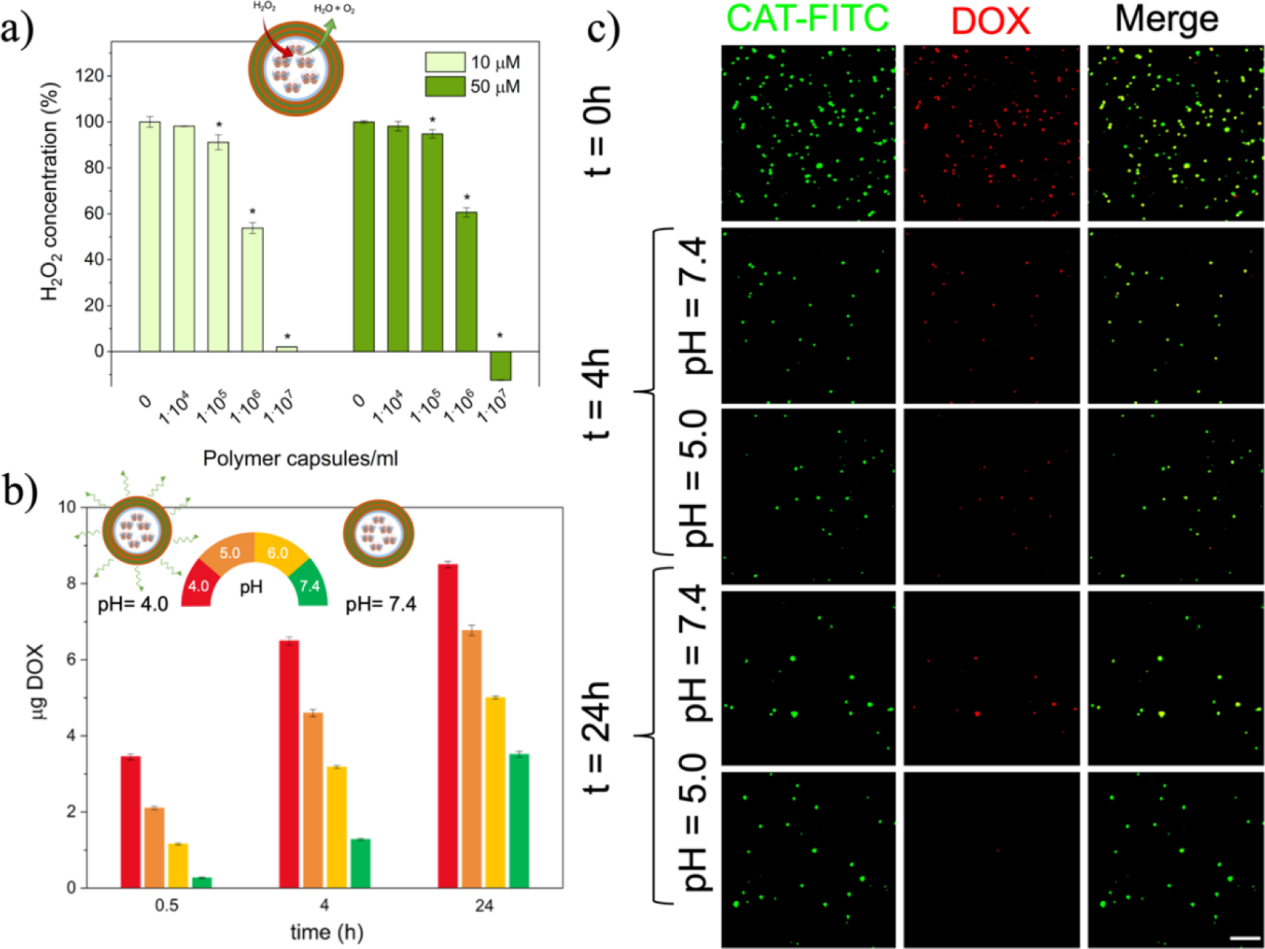
(a) H_2_O_2_ scavenging capacity of polymer capsules at biologically relevant H_2_O_2_ concentrations (10 and 50 μM). Asterisks (*) indicate significant differences (*p* < 0.05) with respect to the control (0 capsules/mL). (b) DOX release at different pH values. (c) Fluorescence micrographs of DOX release at different pH values (FITC-CAT: green/DOX: red). Scale bar: 50 μm.

To assess the antioxidant capacity, two biologically relevant H_2_O_2_ concentrations (10 and 50 μM) were used. Capsules were incubated at a final polymer capsule concentration of 1 × 10^4^, 1 × 10^5^, 1 × 10^6^, and 1 × 10^7^ polymer capsules/mL for 30 min. The concentration of H_2_O_2_ in the solution decreased in a polymer capsule concentration-dependent manner (Figure 3a). At the concentration of 10 μM H_2_O_2_, a significant decrease (*p* < 0.05) in the H_2_O_2_ concentration was observed at the polymer capsule concentrations of 1 × 10^5^, 1 × 10^6^, and 1 × 10^7^ polymer capsules/mL, from the initial value of 100 ± 2.3 to 91.2 ± 3.2, 53.8 ± 2.4, and 2 ± 0.1%, respectively (Figure 3a). In the case of 50 μM, the concentration of H_2_O_2_ decreased significantly (*p* < 0.05) from an initial value of 100 ± 0.6 to 94.8 ± 1.8, 60.6 ± 2, and −12.4 ± 0.2% using the polymer capsule concentrations of 1 × 10^5^, 1 × 10^6^, and 1 × 10^7^ polymer capsules/mL, respectively (Figure 3a). Taken together, the obtained results confirmed the H_2_O_2_ scavenging capacity of the developed polymer capsules, indicating that the activity of the encapsulated enzyme is preserved and the reagents are able to diffuse through the polymeric membrane. The use of antioxidant enzymes is gaining increasing attention for biomedical applications over alternative nonenzymatic antioxidants (*e.g.*, vitamins and flavonoids) thanks to their specificity and efficacy. Furthermore, contrary to nonenzymatic antioxidants, they are not consumed in the reaction with ROS.^57,58^ However, due to their susceptibility to undergo protease degradation and denaturation, several encapsulation strategies [*e.g.*, liposomes,^59^ polymersomes,^60^ and poly(lactide-*co*-glycolide) particles^61^] are being considered. The LbL approach does not require complex chemistries, avoids the use of organic/harmful solvents and conditions, and important aspects of the resulting capsules (*e.g.*, size, shape, and stiffness) can be easily controlled, thus representing a robust strategy over other alternatives.

The scavenging capacity of the encapsulated enzyme after a sterilization process was also evaluated. After submerging the capsules in ethanol (70%), they were incubated with 10 and 50 μM H_2_O_2_ at a final concentration of 1 × 10^6^ and 1 × 10^7^ polymer capsules/mL. Regardless of the H_2_O_2_ concentration, a significant decrease (*p* < 0.05) in their scavenging capacity was observed due to the sterilization with ethanol (Figure S9, Supporting Information). In the case of 10 μM H_2_O_2_ concentration, polymer capsules reduced 94.1 ± 0.6 and 37.9 ± 0.9% of the initial H_2_O_2_ from the solution, using concentrations of 1 × 10^7^ and 1 × 10^6^ polymer capsules/mL respectively, whereas the sterilized counterparts reduced 47.9 ± 2.1 and 0.7 ± 1.2% of H_2_O_2_ (Figure S9, Supporting Information). At the concentration of 50 μM H_2_O_2_, significant differences were also observed in both polymer capsule concentrations. Using 1 × 10^7^ and 1 × 10^6^ polymer capsules/mL, H_2_O_2_ reduction values were determined to be 97.3 ± 2.8 and 35.2 ± 1.8%, respectively, whereas in the case of sterilized capsules, the values were 40.2 ± 4.1, and 18.4 ± 2.4% (Figure S9, Supporting Information). The encapsulation of enzymes into LbL capsules and alternative polymeric systems (*e.g.*, single-enzyme nanogels,^62,63^ polymersomes,^64^ and so on) commonly results in an improved stability of the encapsulated enzyme toward proteolytic degradation, organic solvents, changes in pH and temperature, and so forth.^65,66^ However, in the particular case of LbL capsules exposed to water/ethanol mixtures, an increased permeability has been reported, which is associated to the rearrangement of the polyelectrolytes forming the shell.^67^ This can have a deleterious effect on the catalytic activity of the enzyme and, at the same time, result in its leakage from the polymeric capsule. Therefore, sterilization with ethanol was not considered for the subsequent *in vitro* studies, and alternative approaches to prevent contamination were acquired.

To assess the capacity of the capsules to release a model drug in response to the microenvironment pH, the fabricated polymer capsules were incubated in four different pH buffers (pH = 4.0, 5.0, 6.0, and 7.4). We chose to work with DOX–EMCH as it is a well-established prodrug currently in clinical trials that is stable at neutral pH but undergoes hydrazone cleavage at pH values lower than 6 to release the antiproliferative drug doxorubicin. Supernatants were collected after 30 min, 4 h, and 24 h, and their fluorescence intensities were measured using a microplate reader. After 30 min, the capsules showed an initial release of the model drug of 0.3, 1.2, 2.1, and 3.5 μg at pH = 7.4, 6.0, 5.0, and 4.0, respectively (Figure 3b). As expected, a higher initial release was observed in the more acidic environment. After 24 h, polymer capsules released a total DOX amount of 3.5, 5.0, 6.8, and 8.5 μg at pH = 7.4, 6.0, 5.0, and 4.0, respectively, thus confirming the pH-dependent release of the fabricated polymer capsules (Figure 3b). In comparison to other LbL capsules that rely on a diffusion process to release the encapsulated cargo, our approach allows the delivery of the model drug (*i.e.*, DOX) mainly in acidic conditions. This is of high relevance for the potential translation of this system to biomedical applications, as the off-target effects of the administered drug would be minimized. Diffusion-mediated drug release, apart from being nonspecific to any biologically relevant stimulus, is usually accompanied by an initial burst release. For example, around 80% of the encapsulated DOX was released from capsules made out of PAH/PSS multilayers in less than 300 min^68^ In another example using PSS and poly(amidoamine) dendrimer to fabricate hollow polymer capsules, capsule degradation and/or high ionic strengths were needed to ensure the complete release of the encapsulated DOX.^69^

The pH-dependent release of the fabricated polymer capsules was further confirmed by means of fluorescence microscopy. For this purpose, CAT was stained with FITC (CAT-FITC) prior to the co-precipitation process. After the LbL process and template removal, the capsules were immersed in two of the buffers mentioned above (pH = 5.0 *vs* pH = 7.4), and fluorescent micrographs were acquired after 4 and 24 h. Most of the polymer capsules immersed in pH 7.4 buffer maintained their fluorescence intensity at the assessed time points (4 and 24 h) in both channels (red and green), compared to the capsules before their immersion (0 h) (Figure 3c). In contrast, polymer capsules immersed in the acidic pH buffer were able to emit fluorescence signal only in the green channel (CAT-FITC), but the fluorescence signal in the red channel (DOX) was not visible after 24 h, suggesting a substantial release of the drug (Figure 3c). Although the drug seems to be completely released, the stability of the polymer capsules immersed in pH = 5.0 buffer was preserved, keeping their spherical shape and integrity. Several studies have employed LbL capsules as delivery vehicles for the release of DOX and other therapeutic agents. Most of them rely on the Fickian diffusion release mechanism, where the diffusion is governed by the concentration gradient between the two sides of the polymeric shell, which acts as a barrier. Accordingly, tuning the thickness of the polymeric shell (*e.g.*, by changing the number of deposited layers^68^) or its density (*e.g.*, by promoting shrinkage of the capsules through a thermal treatment^56^ or cross-linking reactions^70^) has been reported as a valid strategy to control the release kinetics of the encapsulated DOX. In this sense, the release of DOX from polymer capsules made out of PAH and dendritic porphyrin was delayed when the polymer layers were cross-linked *via* the carbodiimide chemistry.^70^ To achieve a more specific release at the site of interest, biologically relevant stimuli have been employed as triggers for the disassembly of the capsules and the subsequent delivery of the cargo. Yan *et al.*(71) fabricated LbL polymer capsules stabilized with disulfide bonds that underwent deconstruction during intracellular trafficking due to the reducing environment, thereby leading to DOX release. The acidic microenvironment has been similarly employed to promote the disassembly of hydrazone-bonded polymer capsules, which resulted in a much faster release of the entrapped DOX in comparison to the one observed at neutral pH.^72^ Contrary to these two last examples, our approach allows the release of DOX in response to a biologically relevant stimulus (*i.e.*, acidic pH) while preventing the disassembly of the capsule, which is a must to preserve the protection of catalase and act as a long-lasting microreactor.

Based on these results, we confirm the multifunctional identity of the fabricated capsules, which are able to simultaneously scavenge H_2_O_2_ from the microenvironment in a dose-dependent manner and release DOX in acidic microenvironments.

### 2.4 Metabolic Activity of HeLa Cells in the Presence of Multifunctional Capsules and Internalization

**Figure 4:**
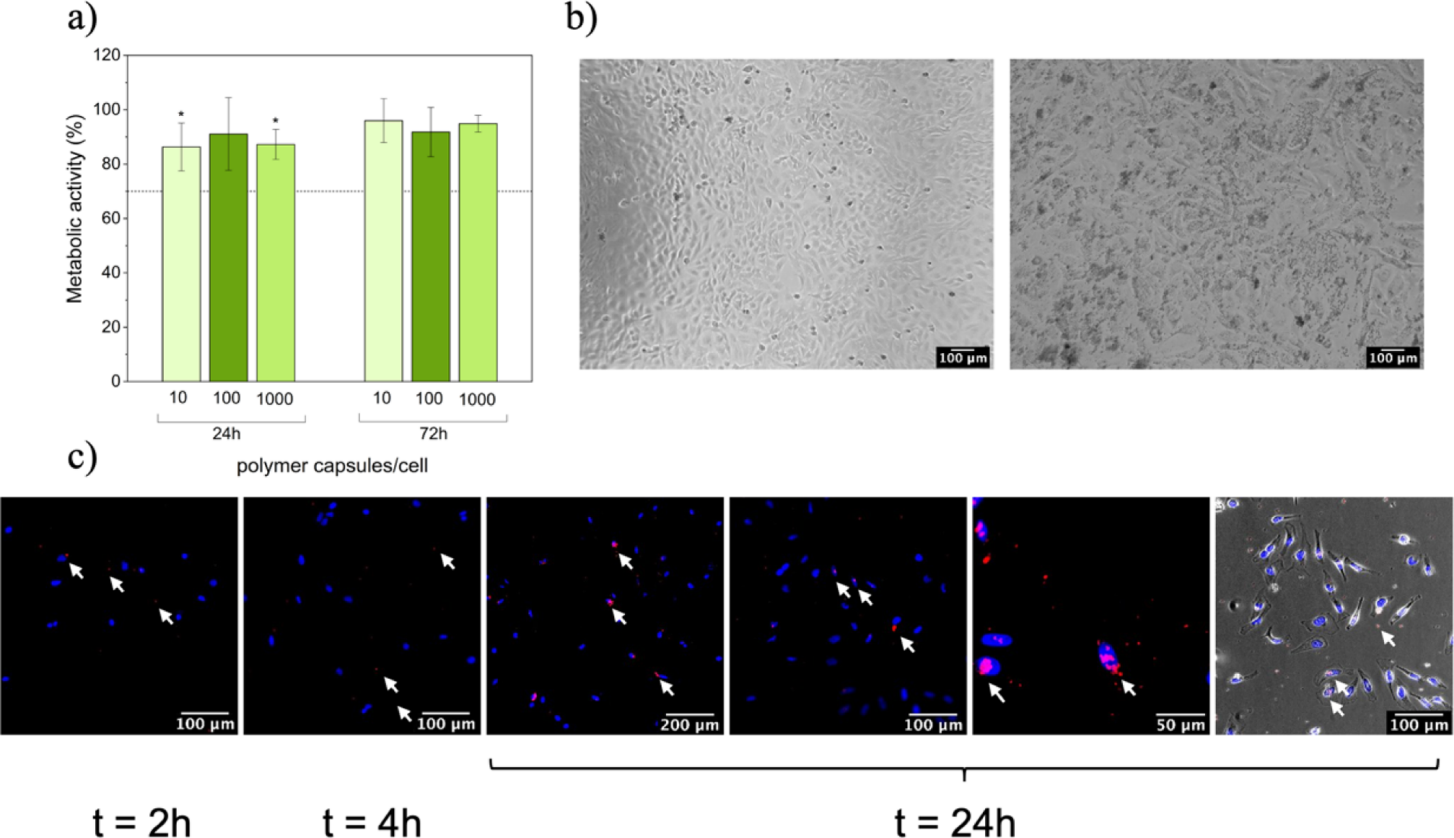
(a) Metabolic activity of HeLa cells in the presence of polymer capsules. Asterisks (*) indicate significant differences (*p* < 0.05) with respect to the control (cells in the absence of capsules), (b) cells in absence of polymer capsules (left) and in the presence of polymer capsules (1000 polymer capsules/cell) (right), (c) fluorescence micrographs of HeLa cells in the presence of polymer capsules at different time points (nuclei-DAPI: blue/polymer capsules functionalized with DOX: red). White arrows highlight the presence of DOX-containing polymer capsules.

The *in vitro* cytocompatibility of polymer capsules [*i.e.*, (PAH/PSS) (dPG-amine/PSS)_2_] was tested with HeLa cells. The metabolic activity of cells in the presence of various capsule per cell ratios (10, 100, and 1000 capsules/cell) was measured after 24 and 72 h by means of AlamarBlue (AB) assay. Cells without capsules were used as a negative control. The cells were able to maintain a normal metabolic activity above the threshold value (*i.e.*, 70%) in the presence of capsules. In fact, after 72 h, no decrease in their metabolic activity (*p* < 0.05) was observed with respect to the negative control (Figure 4a). Although LbL capsules composed of polyelectrolytes are believed to be nontoxic for cells, some studies have reported detrimental effects on cell proliferation and viability at concentrations above 50 capsules per cell.^73^ Besides, the incorporation of inorganic nanoparticles (*e.g.*, magnetic nanoparticles^74^ and manganese dioxide nanoparticles^23^) to provide advanced functionalities also resulted in an increased cytotoxicity of the fabricated capsules in comparison to the nonfunctionalized counterparts, thus reducing the threshold at which these capsules can be employed in the subsequent biological studies. The LbL approach is a highly versatile method that allows to easily tune the surface charge of the resulting capsules. In principle, positively charged capsules improve cell uptake (presumably because of the electrostatic interaction between the surface of the particle and the cell membrane) and can be a valid strategy for some particular applications where internalization is a must (*e.g.*, gene therapy^75^). At the same time, it is generally accepted that positively charged micro- and nanocapsules induce a higher cytotoxicicity.^76^ Based on this “rule of thumb” and on our previous experience,^23^ we engineered the capsules to display an external negative charge. To further confirm the effect of the polymer capsules, cells were observed under an optical microscope. Compared to the negative control (*i.e.*, cells in the absence of polymer capsules), no changes were observed in the morphology of the cells and cell density in the presence of polymer capsules (Figure 4b). Taken together, the results indicate no cytotoxic effect of the fabricated capsules in any of the capsule-to-cell ratios.

The internalization of polymer capsules by both innate immune cells (*e.g.*, macrophages, monocytes, and dendritic cells) and potential target cells must be carefully considered. Phagocytosis by immune cells may be desirable for specific applications, including vaccine carriers. In many other cases (*e.g.*, drug delivery), polymer capsules should escape from the uptake by immune cells to reach the target cells. In any case, the LbL approach offers huge versatility in controlling those key parameters that will determine cell uptake, including size,^77^ shape,^78,79^ surface charge,^80^ stiffness,^81^ and surface chemistry,^53,82,83^ among others. There is a vast literature aimed at unraveling the interplay between these parameters and various internalization mechanisms, sometimes drawing contradictory conclusions. As reported by Novoselova *et al.*,^77^ increasing the size of the LbL capsules from 500 nm to 2 μm reduced the uptake by both macrophages and lung cancer cells from 80 to 20% (approximate values), suggesting that an increased size could be employed as a strategy to avoid macrophage internalization. In another example,^79^ bowl-like microcapsules were preferentially internalized by both smooth muscle cells and macrophages in comparison to spherical counterparts, indicating that isotropic-shaped capsules (*i.e.*, spheres) could be used over anisotropic ones to evade macrophages. However, Shimoni *et al.*(78) reported that capsules with high aspect ratios (*i.e.*, rod-shaped capsules) were poorly internalized by HeLa cells in comparison to spherical ones. This highlights the complexity of endocytosis/phagocytosis processes and their dependence on cell type. Stiffness of the capsules, which can be modulated by the number of layers,^81^ cross-linking reactions, or incorporation of nanoparticles,^84^ also seems to determine the uptake efficiency, the softer capsules with lower stiffness being preferentially internalized by cells with respect to stiffer ones. Tuning the surface chemistry of the capsules can be used to either facilitate or avoid cellular uptake. Engineering the surface of the capsules by attaching bioactive molecules (*e.g.*, peptides, antibodies, and so on) on the outermost layer allows the recognition of receptors and targets the intended cells *via* the antibody–antigen interaction.^82^ Surface PEGylation and similar approaches, in contrast, evade immune clearance and has also been applied to LbL capsules.^83^

Performing a systematic study to analyze the internalization mechanisms of our capsules is beyond the scope of this manuscript. Polymer capsules are transported into cells by endocytosis, but determining the exact mechanism (*e.g.*, phagocytosis, macropinocytosis, caveolae-mediated endocytosis, clathrin-mediated endocytosis, and so on^85^) requires deeper analyses by blocking/inhibiting various cellular endocytic pathways.^79^ Herein, a preliminary study was designed to assess the internalization of the capsules by HeLa cells. Polymer capsules [*i.e.*, (PAH/PSS) (dPG–DOX/PSS)_2_] were incubated at a concentration of 10 polymer capsules/cell and fixed at different time points (2, 4, and 24 h) prior to their observation under a fluorescence microscope. As discussed above, at the selected time points, a negligible release of DOX from the capsules is expected. This was further confirmed in the image merged with the bright field, where DOX was clearly associated to the round-shaped capsules (Figure 4c, right). As observed in Figure 4c, polymer capsules showed a tendency to localize near the nucleus. A progressive accumulation of polymer capsules within the cells was observed along the incubation time and, as a result, most of the capsules were accumulated around the perinuclear region at 24 h. A similar tendency was also reported in the literature, in which a perinuclear accumulation of carriers was observed.^35,36^

### 2.5 Therapeutic Potential of the Polymer Capsules in a H_2_O_2_-Induced Oxidative Stress *In Vitro* Model

Oxidative stress leads to cellular apoptosis and senescence by damaging important cell structures, thus aggravating numerous disease pathologies such as cancer, neurodegeneration, or osteoarthritis. Accordingly, a plethora of biomaterials to control oxidative stress have been developed in the last years including, among others, natural antioxidant-based micro- and nanoparticles (*e.g.*, vitamin-E,^86^ flavonoids,^87^ and so on), synthetic polymeric nanoparticles with intrinsic antioxidant capacity,^88^ and nanozymes based on cerium oxide,^21^ manganese dioxide,^89^ or carbon derivatives.^90^ Although nanozymes represent a promising inorganic alternative to natural enzymes, showing a unique multienzyme mimetic activity, important challenges need to be addressed in terms of long-term cytotoxicity, biodistribution, *in vivo* uptake, and so forth prior to their translation into biomedical applications.^91^ In our present approach, inspired by the compartmentalization strategies found at the cellular and subcellular levels, we encapsulated an antioxidant enzyme (*i.e.*, catalase) into synthetic polymer capsules, resembling artificial organelles. This strategy provides increased robustness to the system by protecting the fragile enzymes from environmental harsh conditions, extending accordingly the storage time and its resistance to temperature, changes in pH, and so forth,^92^ while maintaining its recycling stability (*i.e.*, capacity to efficiently perform successive batch reactions).^66^ All these benefits, together with their cytocompatibility and the possibility to incorporate complementary entities as described above, make LbL capsules an excellent therapeutic platform to protect cells from oxidative stress.

**Figure 5:**
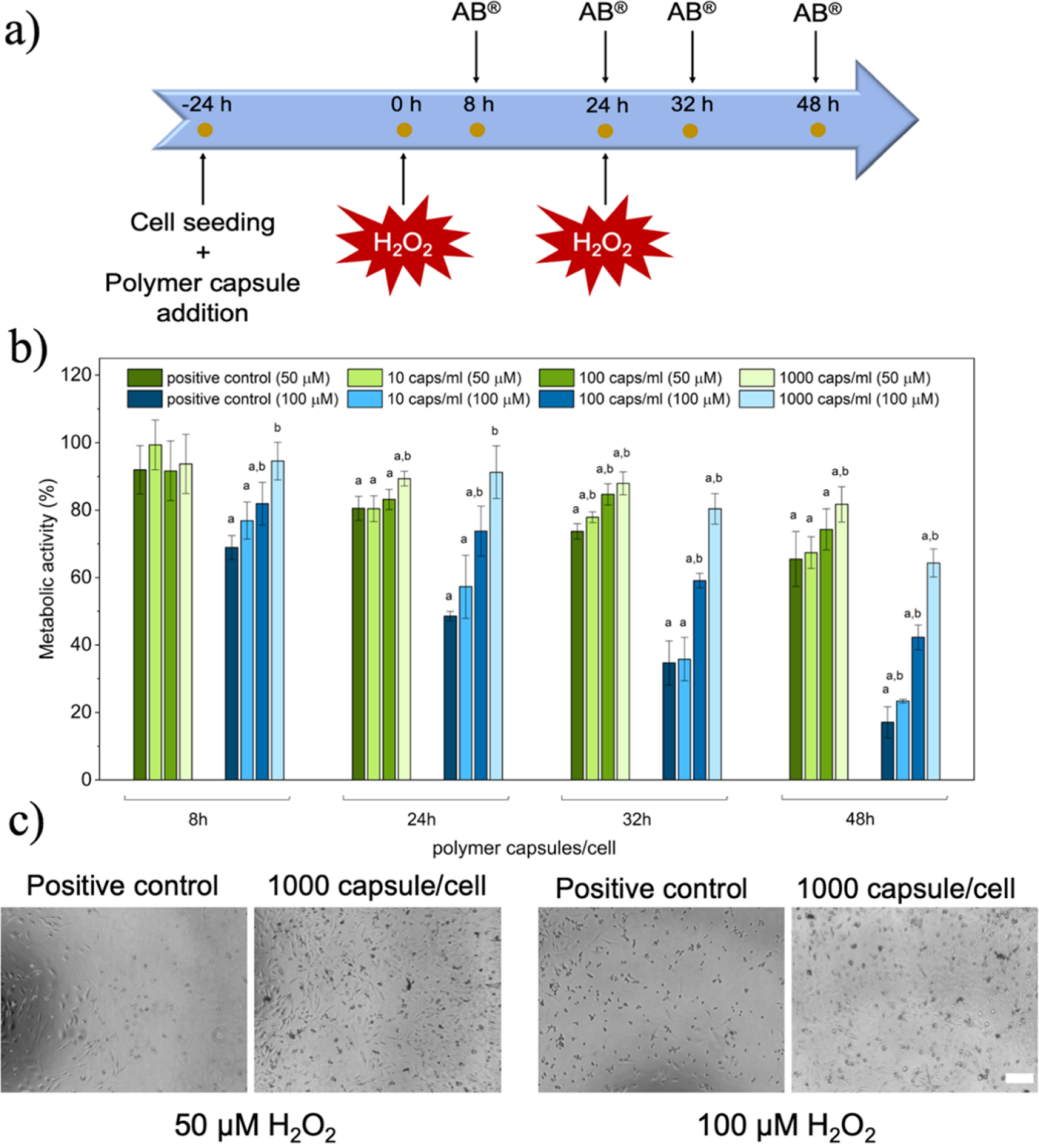
(a) Schematic temporal distribution of stimuli addition and metabolic activity measurements. (b) Metabolic activity of HeLa cells in the presence of H_2_O_2_ stimuli (50 and 100 μM) and polymer capsules; “a” and “b” indicate respectively significant differences with respect to the negative (cells without capsules and H_2_O_2_) and positive controls (cells without capsules but stimulated with 50 or 100 μM of H_2_O_2_) (*n* = 4); 100% of metabolic activity was ascribed to the negative control. (c) Optical micrographs of cells without capsules and stimulated with H_2_O_2_ (positive control) and cells with 1000 polymer capsules/cell and H_2_O_2_ stimuli. Scale bar: 200 μm.

An *in vitro* model with HeLa cells was used to evaluate the therapeutic potential of the fabricated polymer capsules. Cells were stimulated every 24 h with two biologically relevant H_2_O_2_ concentrations (*i.e.*, 50 and 100 μM) to induce oxidative stress (Figure 5a). These H_2_O_2_ extracellular concentrations are assumed to induce deleterious responses on cells, ultimately leading to oxidative distress.^93^ Metabolic activity of the cells was assessed by the AlamarBlue (AB) assay at different time points (8, 24, 32, and 48 h after the initial stimulus) (Figure 5a). The H_2_O_2_ concentrations were chosen after a preliminary analysis to evaluate the effect of different concentrations on metabolic activity (data not shown). HeLa cells in the absence of polymer capsules and H_2_O_2_ stimuli were used as negative control. Cells in the absence of capsules but with H_2_O_2_ stimuli were considered as positive control. The concentrations of polymer capsules employed were 10, 100, and 1000 polymer capsules/cell, and they were not sterilized due to the aforementioned detrimental effect of ethanol on the CAT scavenging capacity (Figure S9, Supporting Information). Alternatively, capsules were fabricated in clean conditions, and all the employed solutions were sterile-filtered. No bacterial or other type of contamination was observed during the course of the experiment. In this particular experiment, capsules without DOX [*i.e.*, (PAH/PSS) (dPG-amine/PSS)_2_] were employed to avoid any possible toxic effect that could mask the protection of capsules against H_2_O_2_-induced oxidative stress.

At a H_2_O_2_ concentration of 50 μM, the metabolic activity of the cells decreased significantly (*p* < 0.05) over time at both the positive control and with 10 polymer capsules/cell, with no significant differences between these two conditions (Figure 5b). This suggested no therapeutic effect of the capsules at this concentration. However, with the addition of higher capsule-to-cell ratios (*i.e.*, 100 and 1000 polymer capsules/cell), a beneficial effect was observed specially with the concentration of 1000 polymer capsules/cell (Figure 5b). At the concentration of 100 polymer capsules/cell, the mean metabolic activity of the cells was always above the positive control, obtaining a significant difference (*p* < 0.05) at 32 h (Figure 5b). At 1000 polymer capsules/cell, significant differences (*p* < 0.05) were observed at all the time points with respect to the positive control, with the exception of the first time point (*i.e.*, 8 h).

Using 100 μM H_2_O_2_ stimuli, a similar trend was observed. In the case of the positive control and 10 polymer capsules/cell concentration, the metabolic activity values were respectively 17.1 ± 4.6 and 23.4 ± 0.6% after 48 h. At 100 and 1000 polymer capsules/cell, the therapeutic effect of the capsules was again validated. With both polymer capsule-to-cell concentrations, the metabolic activity was significantly higher (*p* < 0.05) than in the positive control at all the studied time points. Furthermore, at 1000 polymer capsules/cell, no differences in the metabolic activity were observed in comparison to the negative control (*i.e.*, cells in the absence of polymer capsules and H_2_O_2_) at the first two time points (*i.e.*, 8 and 24 h), and the metabolic activity value was above 80%. These results suggest the therapeutic potential of these capsules to scavenge H_2_O_2_. Although HeLa cells are not representative of any particular disease associated to oxidative stress, their response to H_2_O_2_ is similar to the one observed in other relevant cells. To study the effect of oxidative stress in various diseases (*e.g.*, myocardial infarction, neurodegenerative processes, age-related macular degeneration, and so on), a wide variety of cells (*e.g.*, cardiomyocytes, astrocytes, neural stem cells, and human retinal pigment epithelial cells) have been exposed to H_2_O_2_.^94−97^ In the reported studies, a H_2_O_2_ concentration of 100 μM induced a significant decrease in cell viability. Thus, we believe that the effect of the capsules observed herein with a well-established cell line could be translated to other validated disease models.

To further confirm the obtained results with the AlamarBlue (AB) assay, cells were observed under an optical microscope. In the case of 50 μM of H_2_O_2_, some of the cells in the positive control were dead, whereas in the presence of capsules at a concentration of 1000 polymer capsules/cell, the cells were able to maintain their density with less cell death (Figure 5c), confirming the results obtained with the AlamarBlue assay. In the case of 100 μM of H_2_O_2_, a higher quantity of dead cells was appreciable in the positive control (Figure 5c). Contrarily, cells incubated in the presence of 1000 polymer capsules/cell were alive and maintained their shape and density (Figure 5c).

Taken together, these results confirm the therapeutic effect of the fabricated polymer capsules at the higher capsule-to-cell ratios (*i.e.*, 100 and 1000 polymer capsules/cell), especially with 1000 polymer capsules/cell. At 10 polymer capsules/cell, no therapeutic effect was appreciated, suggesting that the concentration was not enough to protect cells from the H_2_O_2_-induced cell death.

## 3 Conclusions

In this study, we fabricated polymeric capsules *via* the LbL approach and exploited the versatility of this method to incorporate several functionalities into a single polymeric microplatform. The fabricated polymer capsules acted as antioxidant microreactors thanks to the encapsulation of catalase in their core and were able to release a model drug (*i.e.*, DOX) in response to a biologically relevant stimulus (*i.e.*, acidic pH) due to the incorporation of functionalized dPGs in their shell. Contrary to the previously reported delivery systems, our capsules preserve their structural integrity after the drug release process, thus avoiding the leakage of the encapsulated entity and functioning as robust microreactors that perform therapeutic biocatalytic reactions. The cytocompatibility of the developed capsules, which were internalized by cells and preferentially accumulated in the perinuclear region, was confirmed *in vitro*. In our validated oxidative stress model, the use of the higher polymer capsule concentrations resulted in a positive response, showing significant differences in the metabolic activity of the cells in comparison to the positive control (cell without capsules but stimulated with H_2_O_2_). Accordingly, the strategy proposed herein could be used in the development of multifunctional microreactors for the treatment of complex pathologies requiring complementary therapies.

## 4 Materials and Methods

### 4.1 Materials

The following reagents were purchased from Thermo Fisher Scientific: Dulbecco’s modified Eagle’s medium, fetal bovine serum, penicillin–streptomycin, AlamarBlue cell viability reagent, 4′,6-diamidino-2-phenylindole dihydrochloride (DAPI), and 16% formaldehyde solution (w/v). Anhydrous dimethyl formamide (DMF) and anhydrous tetrahydrofuran (THF) were obtained from Scharlab. dPG (MW = 9 KDa, PDI = 1.6 and approximately 121 −OH groups) was prepared according to the published procedure.^98^ The hydrazone derivative of doxorubicin (DOX–EMCH, *i.e.*, DOX bound to 3,3′-*N*-[ε-maleimidocaproic acid]) was prepared as described previously.^99^ The other reagents were purchased from Sigma-Aldrich and used as received. PAH and PSS had molecular weights of *M*_w_ = 17,500 and 70,000 g/mol, respectively.

### 4.2 Synthesis of dPG-Amine

The synthesis of dPG-amine was carried out in the following steps.^100^

#### 4.2.1 Mesylation of dPG

dPG mesylate was synthesized by reacting dPG with mesyl chloride (MsCl). To a solution of dPG in anhydrous DMF, MsCl was added dropwise in an ice bath over a period of 30 min under stirring. The resulting mixture was stirred overnight at room temperature. DMF was removed after 12 h, and the product was dialyzed against the methanol/acetone (70:30) solution for 2 days, changing the solvent twice. The final product, dPG mesylate (dPG-Ms), was obtained as a yellowish oil after the complete evaporation of the solvent with 18 mol % degree of functionalization of mesyl groups.

^1^H NMR (300 MHz, D_2_O): δ 4.2–3.4 ppm (m, 5 H, dPG backbone), δ 3.2 (s, 1 H, CH_3_, OMs) (Figure S1, Supporting Information).

#### 4.2.2 Azidation of dPG

For the synthesis of dPG azide, dPG-Ms was dissolved in anhydrous DMF with the addition of 3 equiv of sodium azide per mesyl group. The mixture was then stirred at 60 °C for 72 h. The resultant solution was cooled down to room temperature and filtered using Celite to eliminate the unreacted sodium azide. The product was then dialyzed against the methanol/chloroform (70:30) solution for 48 h, changing the solvent twice. The final product was obtained with the evaporation of the solvent. The functionalization of dPG with azide groups was confirmed with the complete disappearance of the mesyl (CH_3_) peaks at 3.2 ppm, indicating 15 mol % azide functionalization.

^1^H NMR (300 MHz, D_2_O): δ 4.2–3.5 ppm (m, 5 H, dPG backbone) (Figure S2, Supporting Information).

#### 4.2.3 Amination of dPG

The amination of dPG was carried out by the reduction of azide moieties using triphenyl phosphine (PPh_3_) as the reducing agent. The azide-functionalized dPG was dissolved in water and 4 equiv of PPh_3_ (in THF) per azide group was added twice in a period of 24 h, and the reaction was carried out at 40 °C for 48 h. The resultant solution was filtered to remove PPh_3_ salt and dialyzed against methanol for 48 h, changing the solvent twice. The functionalization of dPG with 15 mol % amine groups was confirmed with NMR.

^1^H NMR (300 MHz, D_2_O): δ 4.2–3.2 ppm (m, 5 H, dPG backbone), δ 2.8–3.2 ppm (m, 1 H, −CH), δ 2.4–2.8 ppm (m, 2H, −CH_2_) (Figure S3, Supporting Information).

### 4.3 Synthesis of dPG–DOX Conjugate

The conjugation of DOX and dPG-amine takes place in two steps in one-pot synthesis. The first step comprises the thiolation of dPG-amine, followed by the conjugation of thiolated dPG with DOX–EMCH using hydrazone bond formation. For the thiolation step, dPG-amine (10 mg/mL) was dissolved in 50 mM sodium phosphate (pH 7.0) containing 5 mM EDTA solution, followed by the addition of a solution of 2-iminothiolane (1.5 equiv per dPG molecule). The mixture was stirred at room temperature for 20 min. After 20 min, a solution of DOX–EMCH (1.2 equiv per dPG molecule) in 10 mM sodium phosphate buffer (pH 5.8) was added to the reaction mixture, and the solution was stirred at room temperature for 2 h. The resultant reaction mixture was concentrated using an Amicon filter (molecular weight cutoff, 3 kDa), followed by purification using Sephadex G-25 column chromatography using 10 mM sodium phosphate buffer (pH = 7). The appearance of a faster band on the Sephadex G-25 superfine column confirmed the conjugate formation. After purification, the conjugate was lyophilized to obtain the product in a dry state.

### 4.4 Physicochemical Characterization of Polymer–Drug Conjugates

NMR spectroscopy was carried out at a frequency of 300 MHz using deuterated water as the solvent for all the samples. The ζ-potential and hydrodynamic sizes were measured on a Zetasizer Nano ZS analyzer using Malvern Instrument. Fresh polymer solutions were prepared at 1 mg/mL in 10 mM sodium phosphate buffer. All measurements were done at 25 °C and pH = 7.4 using a standard rectangular quartz cuvette and for a minimum of 10 runs. FTIR spectroscopy was performed using a Nicolet Avatar 370 operating in attenuated total reflectance (ATR–FTIR). The spectra of the samples before and after the amination of dPG were taken with a resolution of 2 cm^–1^ and averaged over 64 scans. The amount of conjugated DOX to the dPG backbone was determined by measuring the conjugated drug release at pH = 4.0 using UV–vis spectroscopy. All samples were prepared in water of Millipore quality (resistivity 18 MΩ cm^–1^, pH 5.6 ± 0.2).

### 4.5 Fabrication and Characterization of Polymer Capsules

#### 4.5.1 Fabrication of Polymer Capsules

Polymer capsules were fabricated *via* the LbL approach, as previously described.^20^ Na_2_CO_3_ (1 M in distilled water) and catalase (2 mg/mL in Tris-HCl 0.05, pH = 7.0) solutions were poured into CaCl_2_ (1 M in distilled water) solution. After 30 s of stirring at 1100 rpm, the particles were allowed to settle down for 15 min. After this, the particles were collected by centrifugation at 2000*g* and washed (×3) with a 0.005 M NaCl solution. As the CaCO_3_–catalase microparticles have a negative surface charge, PAH [2 mg/mL in 0.5 M NaCl (pH = 6.5)] was used as the first polyelectrolyte. After an incubation time of 12 min, the particles were collected by centrifugation and washed (×3) with 0.005 M NaCl solution. Following the same procedure, the second layer (*i.e.*, PSS) was deposited. After the assembly of the first two polyelectrolyte layers, dPG–DOX conjugate or dPG-amine was used as the positive layer in the following layer depositions, following the same procedure. The particles were resuspended in 2 mg/mL dPG–DOX/dPG-amine solution in 0.5 M NaCl and subsequently washed with 0.005 NaCl. Particles containing six layers were fabricated with a final shell architecture of (PAH/PSS) (dPG–DOX/PSS)_2_ or (PAH/PSS) (dPG-amine/PSS)_2_. To remove the template, the particles were immersed in 0.1 M EDTA solution (three times, 5 min for each incubation).

The successful encapsulation of the enzyme was assessed by using FITC-labeled CAT (CAT-FITC) in the fabrication process. To do so, CAT and FITC at a ratio of 50–100 μg of FITC per milligram of protein were mixed, as previously described by us.^20^

#### 4.5.2 PhysicoChemical and Morphological Characterization of Microcapsules

A scanning electron microscope (Hitachi S-4800) was used to analyze the morphological aspects of the polymer capsules. The microscope was operated at a working voltage of 5 kV and a working current of 10 nA. The ζ-potential was monitored after each polyelectrolyte deposition step by means of a Malvern Instrument Zetasizer (ZEN 3690). A laser scattering particle size distribution analyzer (HORIBA LA-350) provided information about the size distribution of the template. The successful template removal was assessed *via* FTIR spectroscopy (Nicolet Avatar 370), operating in the attenuated total reflectance (ATR–FTIR), as previously described.^23^ To confirm the DOX adsorption, polymer capsules were observed in an inverted fluorescence microscope (Nikon Eclipse Ts2). After the template removal, the capsules were washed thrice with distilled water, and a drop of the solution was taken out and observed under the fluorescence microscope. As a control, polymer capsules fabricated with dPG without the model drug (dPG-amine) were used. The amount of adsorbed DOX was determined by measuring the dPG–DOX concentration in the polyelectrolyte solution before and after each layer deposition. 100 μL samples were taken out from the initial polyelectrolyte solution and from the supernatant of the particle dispersion after the layer incubation. The samples were diluted to 1:5, and the fluorescence intensity (λ_ex_ = 480 nm/λ_em_ = 595 nm) was measured on a microplate reader (BioTek Synergy H1M) to determine the dPG–DOX concentration.

The stability of the fabricated capsules was analyzed by means of SEM. After the template removal, capsules were incubated in PBS at 37 °C, and samples were taken out at different time points (*e.g.*, 4, 24, and 72 h). The images were acquired using SEM, with the same instrument and conditions mentioned above.

The antioxidant capacity of polymer capsules was evaluated using a fluorimetric hydrogen peroxide assay kit (Sigma-Aldrich), as previously described.^23^

The H_2_O_2_ scavenging capacity of the polymer capsules after ethanol sterilization was also determined. Here, sterilized and nonsterilized polymer capsules at 1 × 10^6^ or 1 × 10^7^ polymer capsules/mL were incubated in 10 and 50 μM H_2_O_2_ solutions for 30 min. After the subsequent centrifugation, the H_2_O_2_ concentration in the supernatant was determined, following the procedure described above.

### 4.6 pH-Dependent Drug Release

The release of DOX was performed in the presence of four different buffers. Phosphate buffers (100 mM, pH 6.0 and 7.4) and sodium acetate buffers (100 mM, pH 4.0 and 5.0) were used, and the release study was performed at 37 °C. After their fabrication, the capsules were centrifuged and the supernatant was removed. Then, they were resuspended in 0.5 mL of each buffer and placed in an orbital shaker at 37 °C. At specific time points (30 min, 4 h, and 24 h), the capsule dispersion was centrifuged and the supernatant was collected. After the supernatant removal, the same volume of fresh buffer was added. The fluorescence intensity (λ_ex_ = 480 nm/λ_em_ = 595 nm) of the supernatant was measured on a microplate reader (BioTek Synergy H1M) to determine the released DOX concentration.

DOX release was also assessed qualitatively by the analysis of the decrease of DOX fluorescence intensity. To do so, polymer capsules were fabricated containing CAT-FITC, following the procedure detailed above. The capsule dispersion was split and centrifuged. After this, the two buffer solutions (pH = 5.0 and pH = 7.4) were added and the polymer capsules were incubated at 37 °C. At specific time points (4 and 24 h), the polymer capsules were collected and washed with distilled water to observe them under an inverted fluorescence microscope (Nikon Eclipse Ts2).

### 4.7 *In Vitro* Studies

#### 4.7.1 HeLa Cell Seeding

HeLa cells (ATCC) were seeded, following the same protocol described in our previous publication.^23^ A density of 5000 cells/well on a 96-well plate was used for metabolic activity measurements. A density of 10,000 cells/well on a 24-well plate was used for internalization studies.

#### 4.7.2 Preliminary Cytocompatibility Test

The cytotoxicity of the capsules was evaluated as previously described.^23^ Three capsule-to-cell ratios (10, 100, and 1000 polymer capsules/cell) and two time points (24 and 72 h) were analyzed, and AlamarBlue was used to measure the metabolic activity of cells.

The uptake of the capsules by HeLa cells was also analyzed. Polymer capsules at 10 capsules/cell were incubated with cells during 2, 4, and 24 h. Afterward, the cells were fixed and stained, following the same procedure described before.^23^ The cells were observed under an inverted fluorescence microscope (Nikon Eclipse Ts2).

#### 4.7.3 Therapeutic Potential of the Multifunctional Capsules in a H_2_O_2_-Induced *In Vitro* Model

To assess the capacity of the fabricated capsules to protect cells from a H_2_O_2_-induced oxidative stress, we used our previously reported model.^23^ Three capsule-to-cell ratios (10, 100, and 1000 polymer capsules/cell) were analyzed. Two stimuli of 50 and 100 μM H_2_O_2_ were added at different time points (0 and 24 h). AlamarBlue assay was used to check the metabolic activity of the cells at the selected time points (8, 24, 32, and 48 h).

### 4.8 Statistical Analysis

Data related to the fabrication and characterization of polymer capsules are presented as mean ± standard deviation (SD). In the *in vitro* studies, the results are presented as mean ± SD, with *n* = 4. One-way analysis of variance (ANOVA) was used to test the statistical differences between groups, with the Bonferroni post hoc test and a confidence level of 95% (*p* < 0.05).

## Supporting Information Available

NMR and FTIR spectra of functionalized dPG, size distribution of the CaCO_3_ sacrificial template, FTIR spectra of polymer capsules before and after the template removal, SEM micrographs of polymer capsules at different incubation times, quantification of dPG–DOX incorporation, and catalytic activity of the capsules after sterilization with ethanol (PDF)
