# Supplementary material for: Smart Layer-by-Layer Polymeric Microreactors: pH-Triggered Drug Release and Attenuation of Cellular Oxidative Stress as Prospective Combination Therapy
Source: ACS Appl Mater Interfaces. 2021 Apr 16;13(16):18511–24. doi: 10.1021/acsami.1c01450 (PMC9161222; doi:10.1021/acsami.1c01450)
Supplement: Supplementary file 1 — am1c01450_si_001.pdf [file am1c01450_si_001.pdf]

Supporting information:

Smart Layer-by-Layer Polymeric Microreactors: pH-triggered Drug Release and Attenuation of Cellular Oxidative Stress as Prospective Combination Therapy.

Edurne Marin[a], Neha Tiwari[b], Marcelo Calderón[b,c], Jose-Ramon Sarasua[a], Aitor Larrañaga[a,].*

[a] University of the Basque Country (UPV/EHU), Department of Mining-Metallurgy Engineering and Materials Science, POLYMAT, Faculty of Engineering in Bilbao, Plaza Torres Quevedo 1, 48013 Bilbao, Spain.
[b] POLYMAT, Applied Chemistry Department, Faculty of Chemistry, University of the Basque Country UPV/EHU, Paseo Manuel de Lardizabal 3, 20018 Donostia-San Sebastian, Spain.
[c] IKERBASQUE, Basque Foundation for Science, 48009 Bilbao, Spain.

* Corresponding author. Phone: +0034 946 013935. E-mail: aitor.larranagae@ehu.eus

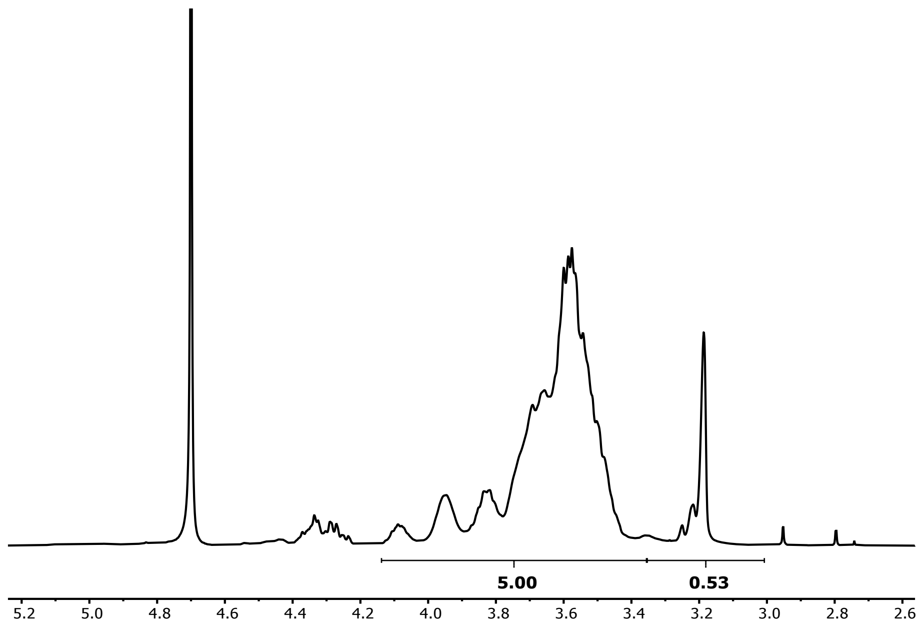

Figure S1. ^{1}H NMR (300 MHz, D_2O) spectra of dPG-mesyl: δ 4.2-3.4 ppm (m, 5 H, dPG backbone), δ 3.2 (s, 1 H, CH_3, OMs)

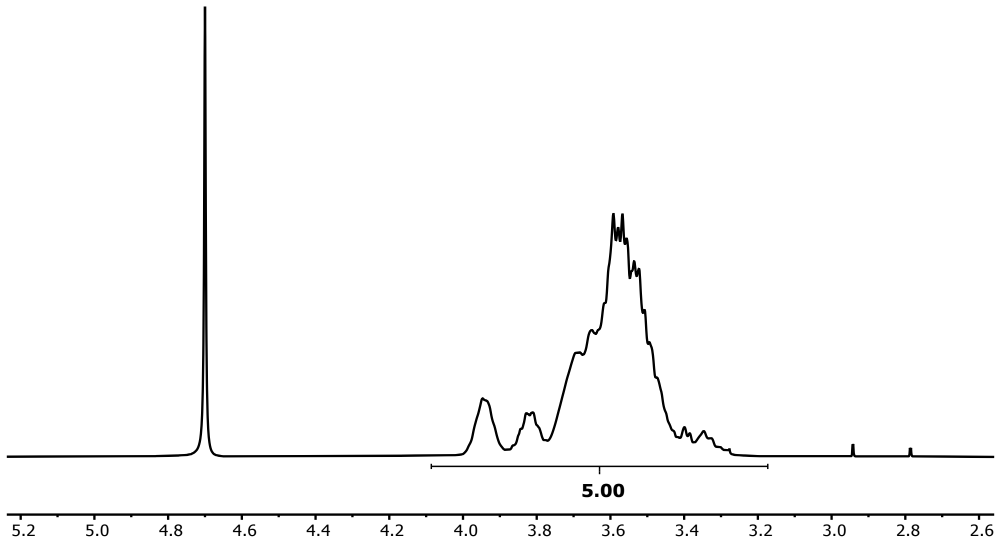

Figure S2. ^{1}H NMR (300 MHz, D_2O) spectra of dPG-azide: δ 4.2-3.5 ppm (m, 5 H, dPG backbone)

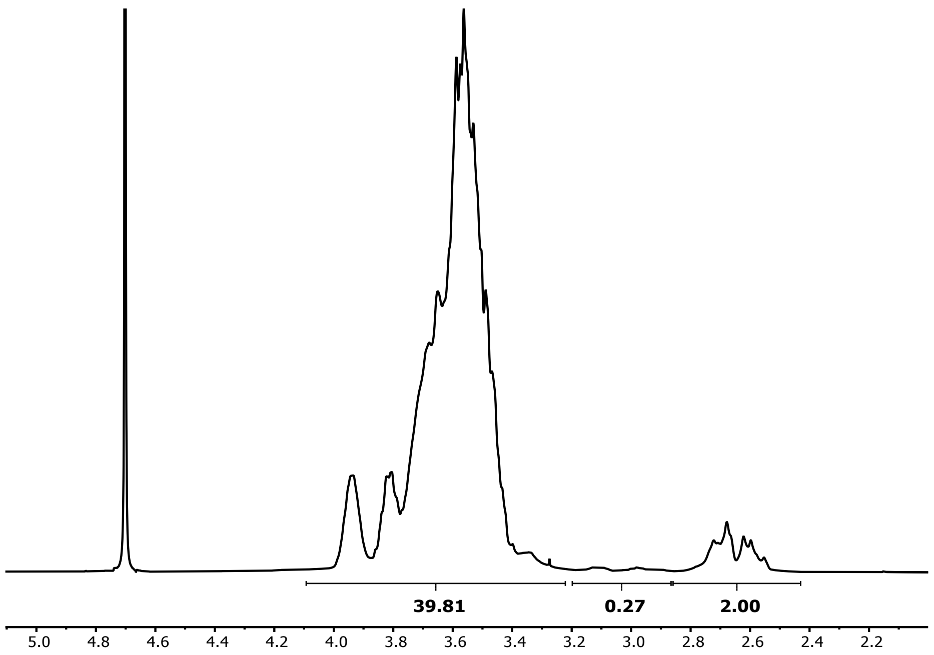

Figure S3. ^{1}H NMR (300 MHz, D_2O) spectra of dPG-amine: δ 4.2-3.2 ppm (m, 5 H, dPG backbone), δ 2.8-3.2 ppm (m,1 H, -CH), δ 2.4-2.8 ppm (m, 2H, $-CH_2$)

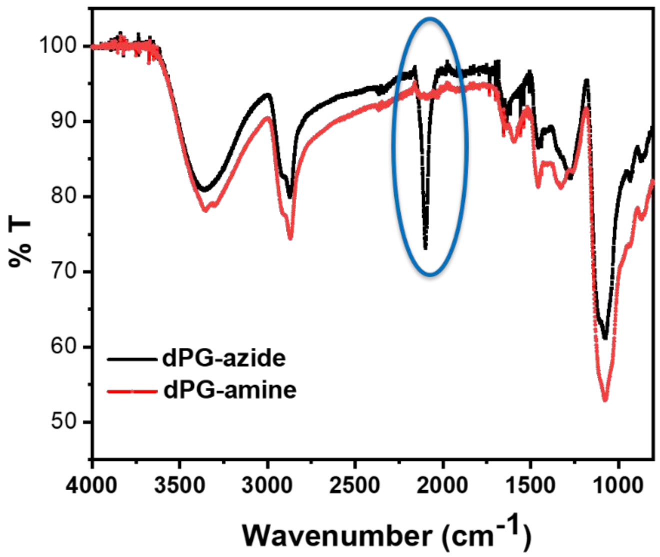

Figure S4. FTIR spectra of dPG-azide (black) and dPG-amine (red). The disappearance of the characteristic peak of azide at 2100 cm^{-1} after reduction to amine is highlighted in the figure.

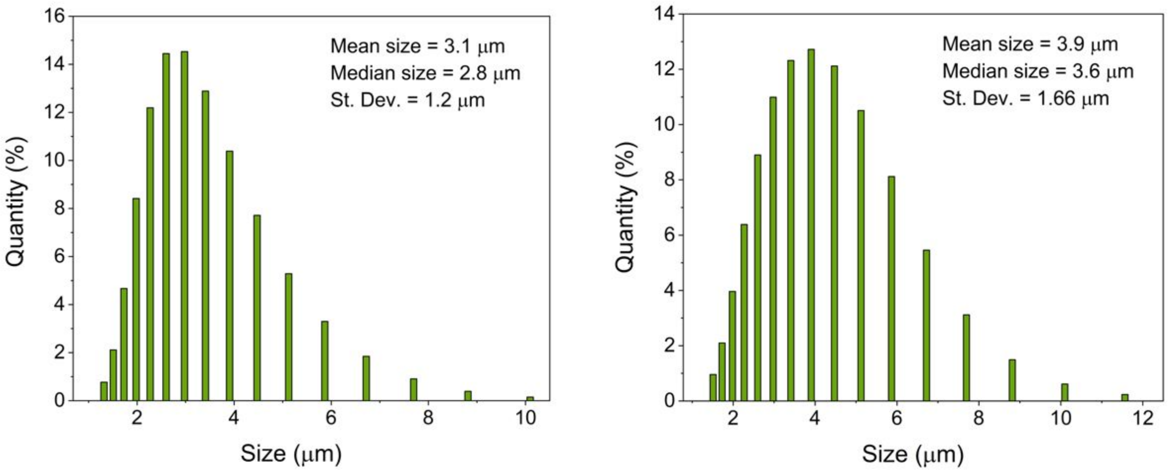

Figure S5. Size distribution of $CaCO_3$ microparticles without (left) and with (right) co-precipitated CAT.

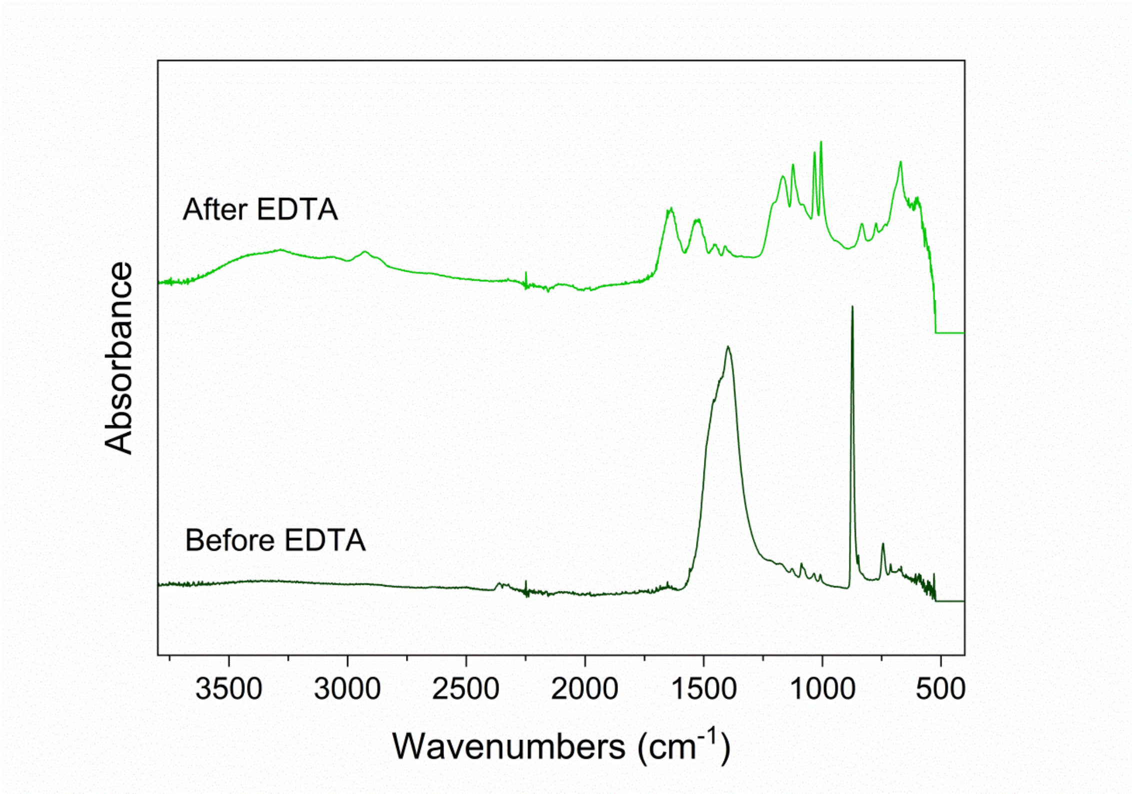

Figure S6. FTIR spectra of polymer capsules before (black) and after (green) template removal.

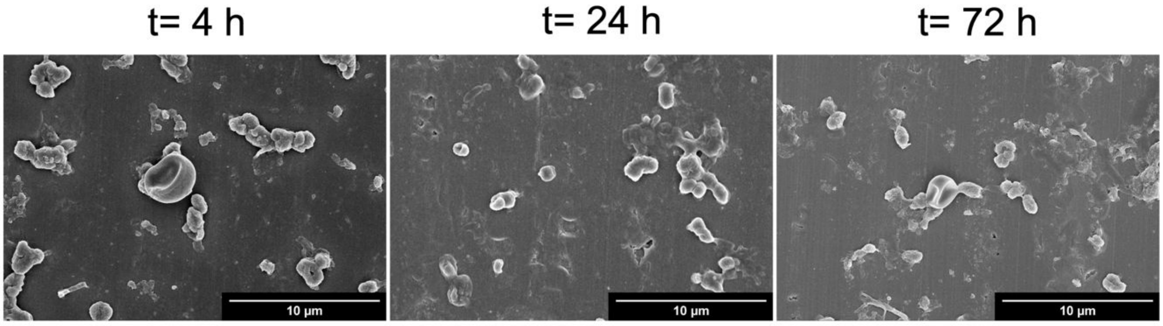

Figure S7. Morphological characterization via SEM of the stability of the polymer capsules over time.

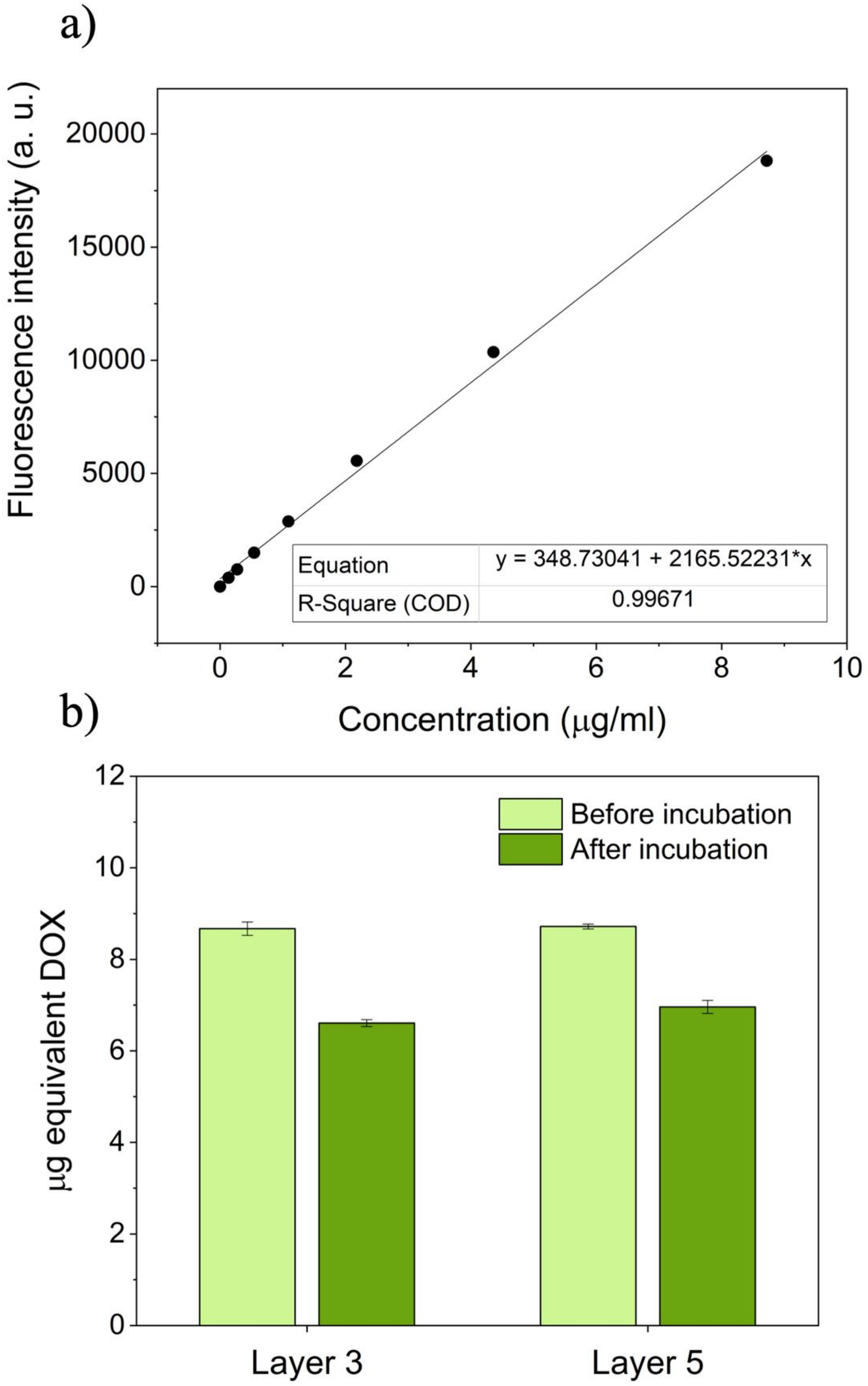

Figure S8. a) Calibration curve of dPG-DOX dissolved in 0.5 M NaCl, b) Adsorbed dPG-DOX in polymer capsules after the 3rd and 5th layer incubation.

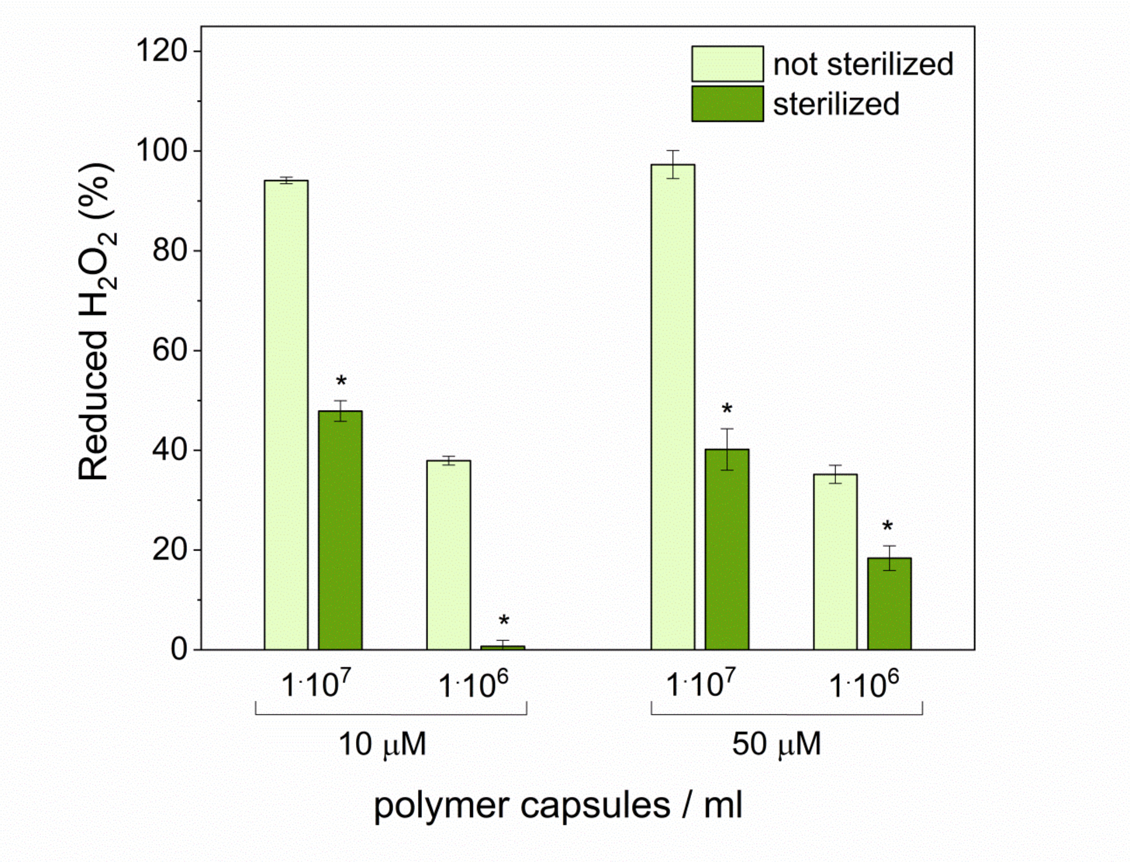

Figure S9. Reduced H_2O_2 by the polymer capsules at 10 μM and 50 μM H_2O_2 after the sterilization process. 100% reduction refers to the complete removal of H_2O_2 from the solution. Asterisks (*) indicate significant differences ($p<0.05$) with respect to the control (non-sterilized capsules).
